# An *in vivo* toolkit to visualize endogenous LAG-2/Delta and LIN-12/Notch signaling in *C. elegans*

**DOI:** 10.17912/micropub.biology.000602

**Published:** 2022-07-28

**Authors:** Taylor N Medwig-Kinney, Sydney S Sirota, Theresa V Gibney, Ariel M Pani, David Q Matus

**Affiliations:** 1 Department of Biochemistry & Cell Biology, Stony Brook University, Stony Brook, NY, USA; 2 current address, Touro College of Osteopathic Medicine, Middletown, NY, USA; 3 Department of Biology, University of Virginia, Charlottesville, VA, USA; 4 Department of Cell Biology, University of Virginia School of Medicine, Charlottesville, VA, USA; 5 D.Q.M. is a paid consultant of Arcadia Science

## Abstract

Notch/Delta signaling regulates numerous cell-cell interactions that occur during development, homeostasis, and in disease states. In many cases, the Notch/Delta pathway mediates lateral inhibition between cells to specify alternative fates. Here, we provide new tools for use in *C. elegans* to investigate feedback between the Notch receptor LIN-12 and the ligand LAG-2 (Delta) *in vivo* . We report new, endogenously tagged strains to visualize LAG-2 protein and *lag-2* transcription, which we combined with endogenously tagged LIN-12 to visualize Notch and Delta dynamics over the course of a stochastic Notch-mediated cell fate decision. To validate these tools in a functional context, we demonstrated that our endogenous *lag-2* transcriptional reporter was expressed in ectopic anchor and primary vulval precursor cells after auxin-mediated depletion of LIN-12. This toolkit provides new reagents for the *C. elegans* research community to further investigate Notch/Delta signaling mechanisms and functions for this pathway *in vivo* .

**Figure 1. A toolkit to simultaneously visualize endogenous Delta and Notch dynamics in vivo .**
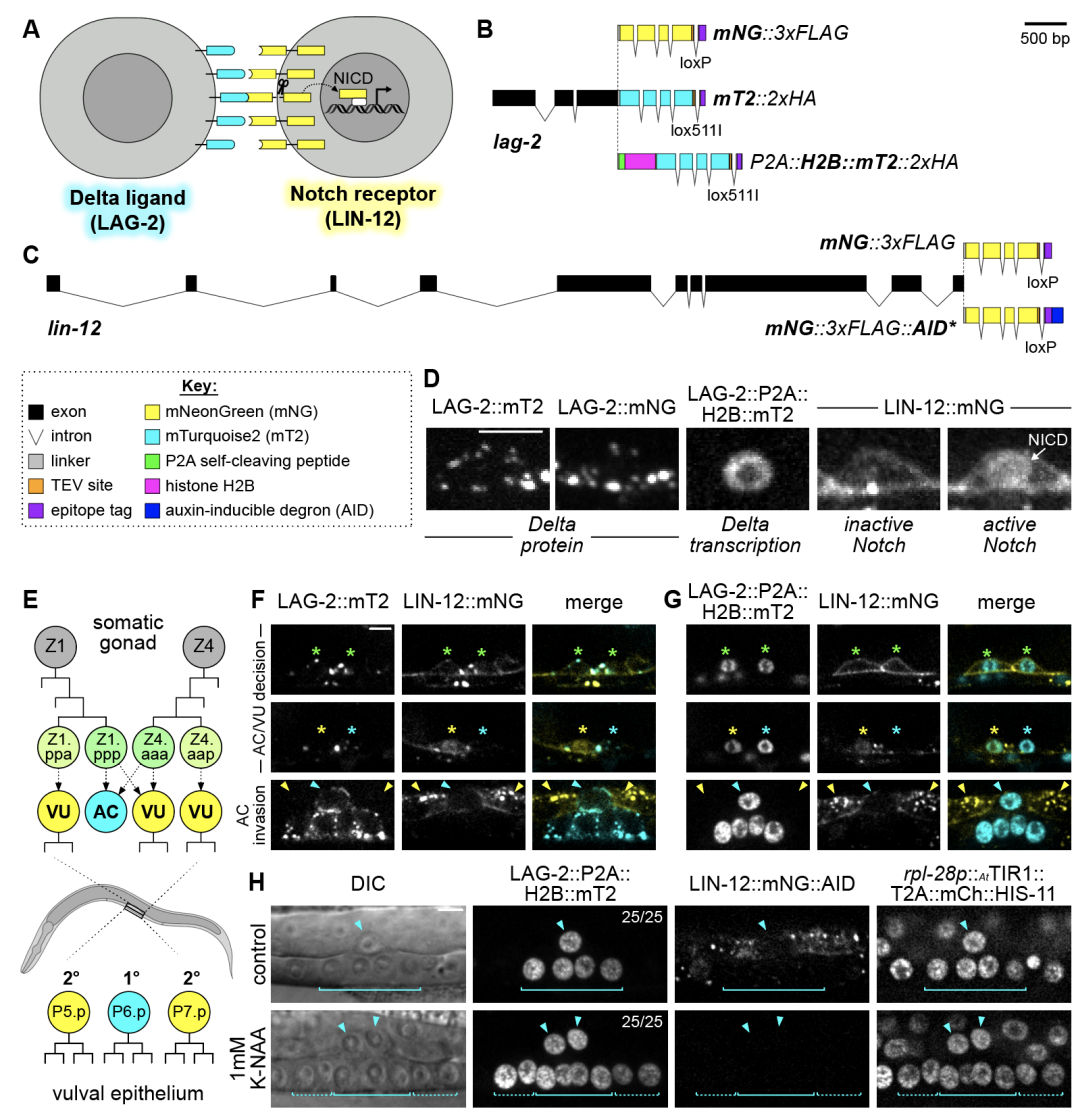
( **A** ) Simplified schematic of Notch-Delta signaling. Binding of the Delta-like ligand LAG-2 (cyan) to the Notch receptor LIN-12 (yellow) results in cleavage of the Notch intracellular domain (NICD) by ɣ-secretase (represented by scissors) and NICD translocation to the nucleus. ( **B-C** ) Schematics of the endogenously tagged *lag-2* ( **B** ) and *lin-12* ( **C** ) alleles used in this study. The *lag-2* transcriptional reporter was generated by including a self-cleaving viral peptide (P2A) and histone H2B (HIS-58) between LAG-2 and mT2 (Ahier & Jarriault, 2014). ( **D** ) Representative micrographs showing localization of the tagged LAG-2 and LIN-12 alleles in anchor cell/ventral uterine cell (AC/VU) precursors. mT2- and mNG-tagged LAG-2 form puncta on the cell membrane while the transcriptional reporter (LAG-2::P2A::H2B::mT2) is nuclear-localized. LIN-12::mNG is expressed on the cell membrane and exhibits nuclear localization of the Notch intracellular domain (NICD) upon activation. ( **E** ) Cartoon of AC/VU cell fate specification (top) as well as primary (1°) and secondary (2°) fated vulval precursor cells (VPCs) (bottom) in a wild-type animal. ( **F-G** ) Micrographs depicting LAG-2::mT2 ( **F** ) or LAG-2::P2A::H2B::mT2 ( **G** ) in concert with LIN-12::mNG reveal Delta and Notch dynamics during the AC/VU decision pre- and post-specification as well as during AC invasion. Asterisks: AC/VU precursors; arrowheads, specified AC and VU cells with colors matching panel **E** . ( **H** ) Endogenous *lag-2* transcriptional reporter expression marks AC and 1° VPC fates in animals harboring LIN-12::mNG::AID and a ubiquitously expressed TIR1::T2A::mCh::HIS-11 transgene. Depleting LIN-12 by treatment with auxin (1 mM K-NAA) leads to specification of a second AC and ectopic 1° VPCs that express LAG-2::P2A::H2B::mT2. Solid bracket, P6.p-derived primary-fated VPCs; dotted bracket, ectopic primary-fated VPCs. Scale bars: 5 µm.

## Description

The Notch signal transduction pathway represents a widely conserved mechanism of cell-cell communication utilized throughout the Metazoa (Gazave *et al* ., 2009). One of the most notable functions for Notch signaling is lateral inhibition, where negative intercellular feedback specifies distinct fates in ligand- and receptor-expressing cells that contact each other (reviewed in Sjöqvist & Andersson, 2019). In a simplified view of Notch signal transduction, Notch ligands and receptors are both transmembrane proteins that activate signaling at cell contacts. The activated Notch receptor then undergoes ɣ-secretase mediated cleavage to release the Notch intracellular domain (NICD), which translocates into the nucleus and acts as a transcriptional co-activator ( **Figure 1A** ) (Struhl *et al* ., 1993). For decades, *C. elegans* has been used as a model investigate functions and mechanisms of Notch signaling. *C. elegans* possesses four canonical Notch ligands in the Delta Serrate LAG-2 family named *lag-2, apx-1, arg-1,* and *dsl-1* (Henderson *et al* ., 1994, Greenwald & Kovall, 2013). *C. elegans* also possesses two Notch receptors, *glp* - *1* and *lin* - *12* , with both unique and redundant functions (Lambie & Kimble, 1991; Greenwald & Kovall, 2013).

To investigate Notch/Delta signaling dynamics *in vivo* , we sought to generate a toolkit to visualize feedback between LIN-12 and LAG-2, which mediates several well-characterized cell fate decisions during larval development (Greenwald *et al* ., 1983; Wilkinson *et al* ., 1994; Levitan & Greenwald, 1998). Because endogenous LIN-12 (Pani *et. al.,* 2022) and LAG-2 (Gordon *et. al.,* 2019) have previously both been tagged with the bright green/yellow fluorescent protein mNeonGreen (mNG) (Shaner *et. al.,* 2013), it is not possible to distinguish them in the same animal using existing tools. To make it possible to visualize endogenously tagged LAG-2 and LIN-12 simultaneously, we tagged LAG-2 with the cyan fluorescent protein mTurquoise2 (mT2) (Goedhart *et. al.,* 2012) ( **Figure 1B,C** ). LAG-2::mT2, and LAG-2::mT2; LIN-12::mNG animals were morphologically indistinguishable from wild-type and did not exhibit the cell fate defects characteristic of *lag-2* or *lin-12* mutants (n ≥ 50 animals examined). Tagged LAG-2::mT2 and LAG-2::mNG protein localized primarily to punctae on the cell surface, which made it challenging to identify the cellular source of expression in contexts where multiple neighboring cells may express LAG-2 ( **Figure 1D** ). To visualize *lag-2* expression with easily discernible cellular resolution, we also generated an endogenous transcriptional reporter by inserting a self-cleaving peptide (P2A) and histone H2B (HIS-58) between LAG-2 and mT2 to generate a bicistronic gene that produces both unlabeled LAG-2 and a nuclear H2B::mT2 marker from the same transcript ( **Figure 1B** ). Unlike traditional transgenic reporters made using somewhat arbitrary promoter fragments, endogenous transcriptional reporters are more indicative of native expression patterns as they reflect the chromatin landscape and full complement of cis-regulatory elements acting on the endogenous gene (Rojas-Fernandez *et al* ., 2015). All of the alleles used here also contain epitope tags (3xFLAG or 2xHA) that can be used for biochemical assays ( **Figure 1B,C** ).

To visualize Notch/Delta signaling dynamics during development, we paired LIN-12::mNG with each of the *lag-2* alleles. These combinations allowed us to visualize LIN-12 activity in concert with LAG-2 protein or *lag-2* transcription during key cell fate specification events *in vivo* . LIN-12 and LAG-2 play important roles in development of the hermaphroditic reproductive system through a lateral inhibition event that specifies distinct fates (Greenwald *et al* ., 1983). In the somatic gonad, specification of the anchor cell (AC) and ventral uterine cells (VU) relies on a stochastic Notch signaling event between two initially equipotent cells, Z1.ppp and Z4.aaa ( **Figure 1E** ). Consistent with expectations, we found that LAG-2 and LIN-12 are first expressed in both AC/VU precursors without visually discernible differences between cells ( **Figure 1F,G** ). During the course of AC and VU cell fate specification, we observed that LIN-12 becomes localized to the nucleus in the presumptive VU where it is activated and is lost from the presumptive AC, while LAG-2 expression becomes restricted to the AC ( **Figure 1F,G** ) (Wilkinson *et al* ., 1994). Although asymmetry in LAG-2::P2A::H2B::mT2 signal is detectable during AC/VU specification, there is a delayed reduction in H2B::mT2 fluorescence that is likely due to perdurance of the histone H2B (Toyama *et al* ., 2013) ( **Figure 1G** ). However, this discrepancy resolves over time, and at later developmental stages we observed strong LAG-2::P2A::H2B::mTurquoise2 signal in the AC with no detectable signal in VU cells ( **Figure 1G** ). This difference in perdurance between proteins suggests a productive approach may be to utilize LAG-2::mT2 to visualize real-time changes in LAG-2 protein levels in parallel with the transcriptional reporter to visualize the identities of LAG-2 expressing cells.

In order to validate our toolkit for reading out molecular phenotypes after functional manipulations, we perturbed Notch signaling by degrading endogenous LIN-12::mNG::AID using the TIR1-AID system (Zhang *et al* ., 2015; Martinez *et al* ., 2020). Degrading LIN-12::mNG::AID (Pani *et. al.,* 2022) using a ubiquitously expressed TIR1 transgene (Hills-Muckey *et al* ., 2022) caused uterine and vulval cell patterning defects consistent with known functions for Notch/Delta-mediated lateral inhibition in AC/VU decision and vulval precursor cell (VPC) fates ( **Figure 1E, H** ) (Greenwald *et al.* , 1983). In control animals, we observed expression of the *lag-2* transcriptional reporter in the single AC and the primary-fated VPC descendants of P6.p. Following treatment with auxin (1 mM K-NAA in solid media) to degrade LIN-12, we observed the phenotype of two ACs and ectopic primary VPCs expressing the *lag-2* transcriptional reporter at 100% penetrance (n = 25) ( **Figure 1H** ). These phenotypes recapitulate those previously reported, where both AC/VU precursors adopt the default AC state, and P5.p and P7.p adopt the primary VPC fate in the absence of Notch signaling (Greenwald *et al* ., 1983).

In summary, here we expand the toolkit for studying Notch/Delta signaling in *C. elegans* by generating additional tools to visualize endogenous LAG-2 protein and *lag-2* transcription *in vivo* . The strains reported here make it possible for the first time to observe endogenous Notch (LIN-12) and Delta (LAG-2) dynamics simultaneously in living animals, which we expect will facilitate increasingly precise efforts to dissect Notch/Delta signaling mechanisms and functions. It is our hope that these reagents will be useful to the *C. elegans* community and others.

## Methods

**Strain maintenance:**

Animals were reared under standard conditions and cultured at either 20°C (for imaging at the L2 stage) or 25°C (for imaging at the L3 stage) (Brenner, 1974). Animals were synchronized through alkaline hypochlorite treatment of gravid adults to isolate eggs (Porta-de-la-Riva *et al.* , 2012). The genotypes of all strains used in this study can be found in the Strain Table.

**CRISPR/Cas9 injections:**

The endogenous *lag-2* locus was edited via CRISPR/Cas9 mediated genome engineering using the self-excising cassette (SEC) method to facilitate screening and the guide RNA targeting sequence 5’ GAAGAATCTAGACATAGTGAC 3’ (Dickinson *et al.* , 2013; Dickinson *et al.* , 2015; Gordon *et. al.,* 2019). The repair templates for *lag-2* were generated by first cloning homology arms, synthesized by PCR using genomic DNA as a template, into pDD315 (mTurquoise2). The LAG-2::mTurquoise2 plasmid was then modified by digestion with the restriction enzyme Bsu36I and assembled with an amplified P2A::H2B sequence through Gibson cloning.

**Auxin-inducible degradation:**

Nematode growth media plates were prepared with the synthetic auxin, K-NAA, for a final concentration of 1 mM, as previously described (Martinez & Matus, 2020). Synchronized L1 stage animals were plated on auxin plates seeded with OP50 *E. coli* .

**Live-cell imaging** :

Micrographs were collected on a Hamamatsu ORCA EM-CCD camera mounted on an upright Zeiss AxioImager A2 with a Borealis-modified CSU10 Yokagawa spinning disk scan head (Nobska Imaging) using 440 nm, 514 nm, and 561 nm Vortran lasers in a VersaLase merge and a Plan-Apochromat 100×/1.4 (NA) Oil DIC objective. MetaMorph software (Molecular Devices) was used for microscopy automation. Animals were mounted into a drop of M9 on a 5% Noble agar pad containing approximately 10 mM sodium azide anesthetic and topped with a coverslip.

**Image processing:**

Images were processed using Fiji/ImageJ (v.2.0.0) (Schindelin *et al.* , 2012). Panels D and H feature maximum intensity projections, whereas panels F and G show single plane micrographs. Schematics of gene loci were generated using sequences from WormBase (Harris *et al.* , 2020) and the Exon-Intron Graphic Maker (http://wormweb.org/exonintron). Figures were assembled in Adobe Illustrator (v.26.0.2).

## Reagents

**Strains:**

**Table:** 

**Strain**	**Genotype**	**Source**
DQM1049	*lin-12* ( *ljf31* [ *lin-12* ::mNeonGreen[C1]^loxP^3xFLAG]) III; *lag-2* ( *bmd204* [ *lag-2* ::mTurquoise2^lox511I^2xHA]) V.	This study
DQM1051	*lin-12* ( *ljf31* [ *lin-12* ::mNeonGreen[C1]^loxP^3xFLAG]) III; *lag-2* ( *bmd202* [ *lag-2* ::P2A::H2B::mTurquoise2^lox511I^2xHA]) V.	This study
DQM1066	*lin-12* ( *ljf33* [ *lin-12* ::mNeonGreen[C1]^loxP^3xFLAG::AID*]) III; *lag-2* ( *bmd204* [ *lag-2* ::mTurquoise2^lox511I^2xHA]) V; *cshIs128* [ *rpl-28p* :: *_At_* TIR1::T2A::mCherry::HIS-11)] II.	This study
DQM1068	*lin-12* ( *ljf33* [ *lin-12* ::mNeonGreen[C1]^loxP^3xFLAG::AID*]) III; *lag-2* ( *bmd204* [ *lag-2* ::mTurquoise2^lox511I^2xHA]) V; *cshIs140* [ *rpl-28p* :: *_At_* TIR1(F79G)::T2A::mCherry::HIS-11] II.	This study
DQM1070	*lin-12* ( *ljf33* [ *lin-12* ::mNeonGreen[C1]^loxP^3xFLAG::AID*]) III; *lag-2* ( *bmd202* [ *lag-2* ::P2A::H2B::mTurquoise2^lox511I^2xHA]) V; *cshIs128* [ *rpl-28p* :: *_At_* TIR1::T2A::mCherry::HIS-11)] II.	Pani *et al.,* 2022
DQM1072	*lin-12* ( *ljf33* [ *lin-12* ::mNeonGreen[C1]^loxP^3xFLAG::AID*]) III; *lag-2* ( *bmd202* [ *lag-2* ::P2A::H2B::mTurquoise2^lox511I^2xHA]) V; *cshIs140* [ *rpl-28p* :: *_At_* TIR1(F79G)::T2A::mCherry::HIS-11] II.	Pani *et al.,* 2022
LP469	*lag-2* ( *cp193* [ *lag-2* ::mNeonGreen[C1]^loxP^3xFLAG]) V.	Gordon *et al.,* 2019

**Plasmids:**

**Table:** 

**Plasmid**	**Description**	**Source**
pAP031	*lag-2* sgRNA plasmid	Gordon *et al.,* 2019
pTNM062	*lag-2* mTurquoise2^SEC^2xHA repair plasmid	This study
pTNM114	*lag-2* P2A::H2B::mTurquoise2^SEC^2xHA repair plasmid	This study
